# High-throughput microRNA sequencing in the developing branchial arches suggests miR-92b-3p regulation of a cardiovascular gene network

**DOI:** 10.3389/fgene.2025.1514925

**Published:** 2025-02-20

**Authors:** Sian Goldsworthy, Marta Losa, Nicoletta Bobola, Sam Griffiths-Jones

**Affiliations:** Faculty of Biology, Medicine and Health, University of Manchester, Manchester, United Kingdom

**Keywords:** branchial arches, GATA6, Tbx20, microRNA, miR-92b-3p

## Abstract

Vertebrate branchial arches (BAs) are a developmental paradigm, undergoing coordinated differentiation and morphogenesis to form various adult derivative tissues. MicroRNAs can strengthen gene regulatory networks (GRNs) to promote developmental stability. To investigate microRNA-mediated regulation in BA development, we generated a novel microRNA-sequencing dataset from mouse BAs. We identified 550 expressed microRNAs, of which approximately 20% demonstrate significant differential expression across BA domains. The three most posterior BAs and the connecting outflow tract (PBA/OFT) express genes important for cardiovascular development. We predicted microRNA-target interactions with PBA/OFT-expressed cardiovascular genes and found target sites for miR-92b-3p to be enriched. We used a dual luciferase assay to validate miR-92b-3p interactions with two transcripts encoding the fundamental cardiac transcription factors (TFs), *Gata6* and *Tbx20*. Furthermore, we demonstrated that miR-92b-3p mimic can downregulate endogenous *GATA6* and *TBX20* in human embryonic stem cells (hESCs) undergoing cardiomyocyte differentiation, confirming microRNA-target binding can occur in a cardiac cell type. miR-92b-3p has previously been shown to target transcripts encoding for two other cardiac TFs, *Hand2* and *Mef2D.* Therefore, we hypothesise that miR-92b-3p acts to stabilise cardiovascular GRNs during PBA/OFT development, through multiple microRNA-mediated regulatory networks.

## 1 Introduction

Vertebrate branchial arches (BAs) represent a principal developmental model that incorporates segmented design, cell migration and tissue specification. The BA transient domains arise during mid-embryonic development and comprise a series of outgrowths on each side of the embryonic head and pharynx, ultimately contributing to mature head, neck, and cardiovascular structures (Frisdal and Trainor, 2014; Xu et al., 2024). Correct formation of these mature structures relies on the interplay between distinct embryonic populations found within the BAs: a mesenchymal core containing mesoderm and cranial neural crest (NC) surrounded by endoderm and ectoderm epithelia (Frisdal and Trainor, 2014; Graham and Richardson, 2012). Cranial NC cells that populate the BAs, originate from the hindbrain, and undergo epithelial to mesenchymal transition to migrate in discrete streams to the BAs (Kulesa et al., 2010). These cells express distinct members of *Hox* cluster genes, which is central to BA patterning (Frisdal and Trainor, 2014). As cranial NC have the potential to give rise to a range of tissues including muscular, skeletal, vascular, and nervous, failure of migration and differentiation has been associated with multiple congenital defects (Etchevers et al., 2019).

In mammals there are five pairs of BAs: BA1, BA2, BA3, BA4, and BA6 (Frisdal and Trainor, 2014). In this study we refer to the latter three, and the connected outflow tract (OFT), as the PBA/OFT. The PBA/OFT domain gives rise to multiple structures of the cardiovascular system, partly due to the migration of a subpopulation of cranial NC called cardiac NC. This subpopulation migrates from more caudal regions of the hindbrain, compared to other cranial NC, into the PBA/OFT (Boot et al., 2003; Kirby et al., 1983; Kirby et al., 1985). Cardiac NC initially form smooth muscle cells for the BA arterial system, connecting the embryonic heart to the dorsal aorta (Bergwerff et al., 1998). BA arteries undergo subsequent remodelling into the great arteries, with cardiac NC contributing to the septation of the OFT into the aorta and pulmonary artery (Hiruma et al., 2002; Jiang et al., 2000). Additional cardiovascular derivatives of cardiac NC cells include parasympathetic nerves and the surrounding cells of the His-Purkinje system (Gurjarpadhye et al., 2007), as well as a reported small proportion of cardiomyocytes (Soldatov et al., 2019). The PBA also contribute to the formation of carotid arteries supplying blood to the head and neck (Frisdal and Trainor, 2014).

MicroRNAs are important factors in regulating embryonic development, with multiple studies validating their roles in cell fate decision and tissue patterning (Aboobaker et al., 2005; Alzein et al., 2021; Crist et al., 2009; Li et al., 2021). Generally, microRNAs are understood to execute two different mechanisms of regulation. The first mechanism regards microRNAs as “on-off” switches, whereby they are anticorrelated in expression with their targets; this mechanism has been associated with earlier embryonic development (Avital et al., 2017). The second mode sees microRNAs as fine-tuners of their targets, stabilising their expression and attenuating noise brought about by stochasticity. These microRNAs are reported to be expressed later during development and generally show overall weaker repressive abilities (Avital et al., 2017). Weaker repression across many targets has been shown to stabilise GRNs through cumulative effects (Chen et al., 2019; Ma et al., 2018; Zhao et al., 2018).

MicroRNA-mediated regulation occurs during cardiovascular and craniofacial development. Disruption of the microRNA biogenesis pathway, through NC conditional *Dicer* loss of function, has been implicated in abnormal BA vessel remodelling (Nie et al., 2011) and OFT morphogenesis (Saxena and Tabin, 2010). Furthermore, *Dicer* loss of function in mouse models resembles several congenital phenotypes observed in DiGeorge Syndrome (DS) patients (Nie et al., 2011). DS individuals commonly have a genomic deletion encoding *DGCR8* (Sellier et al., 2014), which is essential for microRNA processing (Gregory et al., 2004). In addition to incorrect BA arterial remodelling, disrupting microRNA biogenesis also leads to craniofacial defects, due to aberrant skeletal formation and muscular maldevelopment (Nie et al., 2011). Taken together, microRNA biogenesis and expression are important for normal development of BA derivatives.

In this study we present novel microRNA-seq datasets that characterise global microRNA expression during mouse BA development. We find 550 microRNAs expressed across BA1, BA2, and the PBA/OFT, with many microRNAs demonstrating progressive expression across the anterior-posterior axis. Using time-matched BA RNA-seq datasets (Losa et al., 2017), we identified candidate microRNA target genes enriched for biological processes linked to cardiovascular development. Using *in silico* microRNA target prediction and *in vitro* microRNA target validation, we identify a role for miR-92b-3p as a key cardiovascular developmental regulator, adding to previous studies that have demonstrated miR-92b-3p regulates transcripts that encode for cardiac TFs *Hand2* and *Mef2d* (Chen et al., 2012; Hu et al., 2017; Yu et al., 2019). We hypothesise that miR-92b-3p works in multiple microRNA-mediated coherent feedforward loops within cardiovascular GRNs, ultimately stabilising target gene expression during mammalian PBA/OFT development.

## 2 Materials and methods

### 2.1 BA dissection, RNA extraction and library preparation

Wild type (CD1) mice were time-mated to obtain tissue for microdissection. Animal experiments followed local legislations regarding housing, husbandry, and welfare (ASPA 1986; United Kingdom). Embryos were collected at E10.5 and E11.5 and accurately staged by counting somites. BA1, BA2 and PBA/OFT tissues were dissected and snap frozen on dry ice and stored at −80°C until RNA extraction. The OFT was harvested with the PBA as it acts as a landmark feature during dissection and maintains physical integrity of the PBA. BAs from three closely staged embryos were pooled for each library, with BA1, BA2 and PBA/OFT from the same embryos being used for each timepoint replicate. Total RNA was extracted using the miRNeasy micro kit (Qiagen, #217084) following the manufacturer’s instructions, eluted into RNase-free water and stored at −20°C until library preparation. Small RNA libraries were generated using the NEBNext Small RNA Library Prep Set (New England BioLabs, #E7330S) following manufacturer’s instructions. For size selection we used gel separation and extracted amplified microRNA cDNA bands corresponding to 140bp. NEBNext Index primer sequences and respective libraries are listed in Supplementary Table S1. Libraries were quality checked using the Agilent 2200 BioAnalyzer TapeStation and sequenced on the Illumina HiSeq 4000 at The University of Manchester Genomics Technologies Core Facility. Small RNA-seq libraries have been deposited under project accession PRJEB64007 available from the European Nucleotide Archive.

### 2.2 Small RNA-seq analysis

NEBNext Small RNA adapter sequences were removed using cutadapt v1.8 (Martin, 2011). Adapter-trimmed reads were filtered to keep those 18–25 nt in length, and mapped against mouse tRNAs [mm 10, GtRNAdb v18.1 (Chan and Lowe, 2016)] and rRNAs [*Mus musculus*, Silva SSU/LSU r138.1 (Ludwig et al., 2004; Quast et al., 2013)], using Bowtie v1.1.0 (Langmead et al., 2009). Reads that mapped to tRNAs/rRNAs were discarded from further analysis. Remaining reads were mapped to the mm10 primary fasta file (GRCm38, release M23) using bowtie v1.1.0 (Langmead et al., 2009) with the following settings: bowtie -v1 -a -m5 –best–strata. To predict novel microRNAs, we used miRDeep2 v0.1.3 (Friedländer et al., 2011) with combined filtered reads from all our small RNA-seq libraries. Reference microRNAs included stem-loop mouse, mature mouse and mature rat microRNAs, all downloaded from miRBase v22 (Kozomara et al., 2019). Novel pre-microRNAs were filtered using the following criteria: ≥30 0-mismatch reads for the mature arm and ≥10 0-mismatch reads for the star arm, no internal sub-hairpins, ≥50% 5′ arm homogeneity, 0-4 nt overhang at the 3′ arm, hairpin free energy ≤ −0.2 kcal/mol/nt. Custom Python scripts used for filtering are available at github.com/SianGol. Pre-microRNA sequences that met all the above criteria were input into Rfam v14.6 sequence-search (Kalvari et al., 2020) to remove any that overlapped with previously annotated ncRNAs. Remaining novel microRNAs were added to the GTF file of known microRNAs, downloaded from miRBase v22 (Kozomara et al., 2019). Genome-mapped reads were assigned to mature microRNAs and quantified using featureCounts v1.6.0 (Liao et al., 2014) with the following settings: featureCounts -M -g gene_id -s 0.

### 2.3 RNA-seq analysis

BA1, BA2 and PBA/OFT E10.5 and E11.5 (Losa et al., 2017) RNA-seq libraries were adapter-trimmed and quality-filtered using Trimmomatic v0.36 (Bolger et al., 2014). We used STAR v2.5.3a (Dobin et al., 2012) to generate the mm 10 genome index (GRCm38, release M23) and map RNA-seq reads to the genome using the mm 10 genome primary fasta file and corresponding GTF file, both downloaded from GENCODE (Frankish et al., 2021). Mapped reads were then assigned to annotated genomic features and quantified using featureCounts v1.6.0 (Liao et al., 2014), with options set as following: featureCounts -g gene_id -s 2.

### 2.4 Differential expression of small RNA and RNA-seq datasets

To remove lowly-expressed genes, a threshold was set for our RNA-seq libraries: 4.2CPM (corresponding to approximately 100 reads) for small RNA-seq, and 0.38 CPM (corresponding to approximately 10 reads) for RNA-seq. Genes with CPM above these thresholds in two or more libraries were kept for further analysis. Differential analysis was performed using DESeq2 (Love et al., 2014) and results were obtained for the following pairwise comparisons: E10.5 BA1 vs. BA2, E10.5 BA1 vs. PBA/OFT, E10.5 BA2 vs. PBA/OFT, E11.5 BA1 vs. BA2, E11.5 BA1 vs. PBA/OFT, E11.5 BA2 vs. PBA/OFT.

### 2.5 MicroRNA target prediction

3′UTR coordinates of BA transcripts were identified using the mm10 GTF and extract_transcript_regions.py (Floor, 2018). The longest 3′UTR for each gene was used, alongside BA expressed microRNAs, as inputs for *in silico* microRNA target prediction by seedVicious v1.3 (Marco, 2018). Results were filtered to remove predicted interactions with 6mers, off-6mers, sites with a hybridisation energy of >−7 kcal/mol, and 3′UTRs with <2 predicted microRNAs binding sites.

### 2.6 PBA/OFT gene set and microRNA-target enrichment analysis

E10.5 PBA/OFT Gene Ontology (GO) terms were identified using PANTHER (Mi et al., 2018). The background genes used were those differentially expressed (±1.5-fold, adj-p ≤ 0.05) in any BA pairwise comparison, and the input genes were those >1.5-fold (adj-p ≤ 0.05) expressed in the PBA/OFT compared to BA1 and BA2. We defined our PBA/OFT gene set as those annotated under the most significant GO terms returned (≥2-fold, FDR ≤ 0.05) by PANTHER. To perform a hypergeometric test for enrichment, we used the phyper function in R, with PBA/OFT-gene-set-microRNA interactions as the ‘sample’, and all BA-microRNA interactions as the “population”.

### 2.7 hESC cardiomyocyte differentiation

NKX2-5^eGFP/w^ hESCs (Elliott et al., 2011) were seeded in hESC medium (DMEM/F-12 (Gibco, #31765027), 1X None-Essential Amino Acids (Gibco, #11140050), 1X GlutaMAX (Thermo Scientific, #35050038), 0.1 mM 2-ME (Gibco, #21985023), 0.5% penicillin-streptomycin (Sigma-Aldrich, #P0781), 20% KnockOut Serum Replacement (KSR) (Gibco, 10828028), and 10 ng/mL bFGF (Miltenyi, #130-104-924)) at a density of 1.8 × 10^5^ cells/mL on growth factor-reduced Matrigel coated 6-well plates. Twenty-four hours later (day 0) differentiation was induced as described previously (Giacomelli et al., 2020). hESC medium was replaced with BPEL (Ng et al., 2008) supplemented with BMP4 (Bio-techne/R&D, #314-BP-050), 20 ng/mL ACTIVIN A (Miltenyi Biotec, #130-115-009) and 1.75 μM CHIR99021 (Selleckchem, #S1263). On day 3, media was refreshed with BPEL containing 1 μM XAV939 (VWR, #CAYM13596-1). BPEL was refreshed every 3 days thereafter.

### 2.8 Cloning 3′UTRs into dual luciferase reporter vectors

Candidate target 3′UTRs were amplified using the following reaction; 1X Q5 Reaction Buffer (New England BioLabs, #B9027S), 20 units/mL Q5 High-fidelity DNA Polymerase (New England BioLabs, #M0491S), 0.6 μM 3′UTR F/R primer (Supplementary Table S1), 0.2 mM dNTP mix (Promega, #U1511), 2 μL DNA, and RNase free water, using the cycling conditions: 98°C 1 min, 35X [98°C 10 s, 58°C 30 s, 72°C 1 min], 72°C 2 min. Amplified 3′UTRs were size selected and purified using the QIAquick Gel Extraction kit (Qiagen, #28704) following manufacturer’s instructions. Purified 3′UTRs were ligated with the pmirGLO dual-luciferase vector (Promega, #E1330) via SacI and SalI restriction sites. Resultant plasmids were sequenced using 0.4 μM pmirGLO F/R custom sequencing primers (Supplementary Table S1).

### 2.9 MicroRNA 3′UTR binding site mutagenesis

miR-92b-3p binding sites in each 3′UTR were mutated using the QuikChange II XL site-directed mutagenesis kit (Agilent, #200517) following the manufacturer’s instructions, using custom mutagenesis primers (Supplementary Table S1). Mutated plasmids were sequenced as described above.

### 2.10 Dual luciferase reporter assay

NIH/3T3 cells were reverse co-transfected with 100 ng pmirGLO dual luciferase vector (Promega, #E1330) containing appropriate 3′UTRs and 30 nM microRNA mimic in 96-well plates (Invitrogen, miR-92b-3p #4464066, miRNA negative control 1 #4464058) using lipofectamine 2000 (Invitrogen, #11668019) diluted in opti-MEM (Gibco, #31985062). Luciferase activity was measured 48 h later using the Dual-Glo Luciferase Assay System (Promega, #E2920) following the manufacturer’s instructions. Firefly and Renilla luciferase signal were measured using the Promega GloMax-Multi + Detection System. Five technical replicates were performed for each biological replicate. Firefly luciferase values were first normalised to Renilla luciferase, and fold change was calculated relative to a control sample transfected with the dual-luciferase plasmid and no microRNA mimic.

### 2.11 miR-92b-3p mimic transfection

HEK293 and NKX2-5^eGFP/w^ hESCs were reverse transfected with 30 nM microRNA mimic (Invitrogen, miR-92b-3p #4464066, miRNA negative control 1 #4464058) using lipofectamine 2000 (Invitrogen, #11668019) diluted in opti-MEM (Gibco, #31985062). Samples were incubated for 24 h at 37°C in 5% CO2.

### 2.12 RNA extraction and RT-qPCR

Where hESC cardiomyocyte differentiation timeline samples were used for microRNA and mRNA RT-qPCR, total RNA was extracted using the mirVana miRNA Isolation kit (Invitrogen, #AM1560) following the manufacturer’s instructions. Alternatively, where only mRNA expression was measured, RNA was isolated using TRIzol Reagent (Invitrogen, #155996026) following a standard protocol. For miR-92b-3p quantification, 10 ng total RNA was used as input with the TaqMan Advanced MiRNA cDNA Synthesis Kit (Applied Biosystems, #A28007) following the manufacturer’s instructions. For our internal control, U6, 10 ng total RNA was used as input for the TaqMan MicroRNA Reverse Transcription Kit (Applied Biosystems, #4366596) following manufacturer’s instructions. To measure miR-92b-3p and U6 expression we used the TaqMan Fast Advanced Master Mix Kit (Applied Biosystems, #4444556) according to manufacturer’s instructions, with 1X TaqMan Advanced miRNA Assay (Applied Biosystems, #A25576, assay ID: 477823_mir) or 1X TaqMan small RNA Assay (Applied Biosystems, #4427975, assay ID: 001973) respectively. For mRNA expression we used the QuantiTect SYBR Green RT-PCR kit (Qiagen, #204243) in the following reaction: 1X QuantiTect SYBR Green RT-PCR master mix, 0.3  μM F/R primers (Supplementary Table S1), 1X RT mix, 40 ng RNA, RNase-free water. Cycling conditions: 50°C 30 min, 95°C 15 min, 40X [95°C 20 s, 57°C 30 s, 72°C 30 s], 68°C 7 min, 4°C hold. Fold change was calculated using 2^−ΔΔCt^ relative to the given control.

### 2.13 Western blotting

HEK293 cells were lysed (20 mM Tris-HCl, 120 mM NaCl, 0.5 mM EDTA, 0.5% NP40 (Thermo Scientific, #J60766-AP), 10% glycerol (Thermo Scientific, #17904), 1X protease cocktail inhibitor (Roche, #4693116001)) and the supernatant was recovered. Proteins were denatured in 1X laemmli buffer. Western blot membranes were incubated with 1:1500 anti-GATA-6 rabbit-mAb DEIE4 (Cellsignal, #5851) or 1:50,000 anti-β-Actin-peroxidase mouse-mAb (Sigma Aldrich, #A3854) in 1% milk. For GATA6 1:10,000 Goat anti-Rabbit IgG HRP (abcam, #ab6271) was used as a secondary antibody in 1% milk.

### 2.14 Sequence alignment

miR-92 sequences for human, mouse, zebrafish and *Drosophila* were obtained from miRBase v22 (Kozomara et al., 2019). *Gata6* and *Tbx20* 3′UTR sequences were obtained from the UCSC genome browser versions hg38 and mm 10. To align sequences, we used Clustal Omega (Sievers and Higgins, 2014).

## 3 Results

### 3.1 BAs demonstrate distinct microRNA expression patterns

To identify microRNAs that function during development and morphogenesis of mammalian BAs, we generated small-RNA-seq libraries for BA1, BA2, and PBA/OFT tissue from embryonic (E) 10.5 and E11.5 mouse embryos. Both timepoints coincide with mid-gestation and allow us to capture the three separate BA domains prior to their fusion at E11.5-E12.5 (Frazer, 1926). Furthermore, these datasets complement RNA-seq libraries we previously generated from equivalent tissues and timepoints (Losa et al., 2017), and so provide a valuable opportunity to consider expression of both microRNAs and predicted target mRNAs.

**FIGURE 1:**
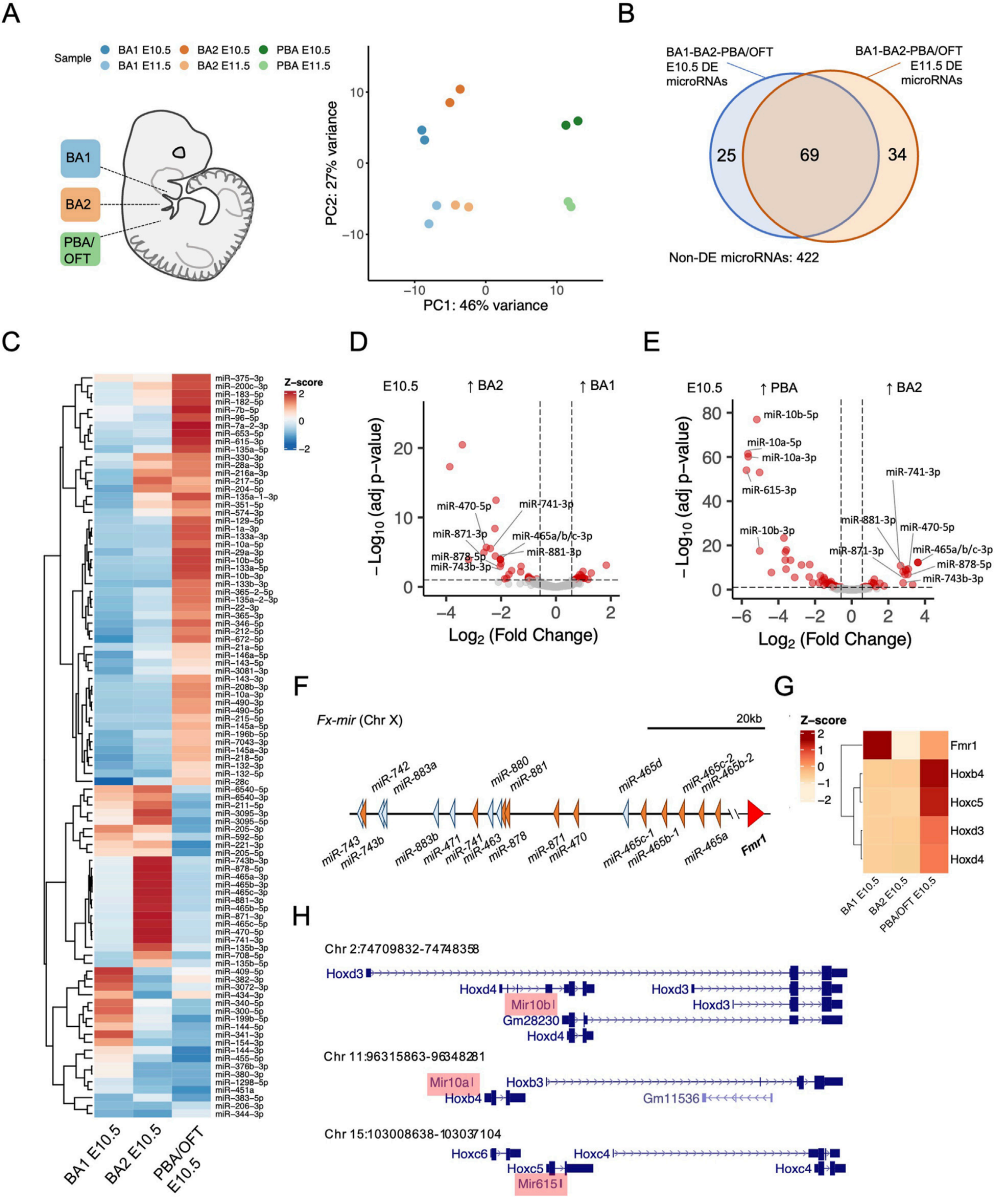
Differential expression of microRNAs across mouse branchial arches. **(A)** Principal component analysis of branchial arch microRNA-seq libraries, mapped to the mm10 genome and assigned to mature microRNAs. **(B)** Overlap of differentially expressed microRNAs (≥1.5-fold change and adj-p ≤ 0.05), from BA pairwise comparisons at E10.5 and E11.5. **(C)** Z-score normalised expression of differentially expressed microRNAs from E10.5 BA pairwise comparisons. **(D, E)** Pairwise comparisons between E10.5 BA1 v BA2 and E10.5 BA2 v PBA/OFT. Fx-mir and Hox cluster microRNAs are labelled. Dotted lines correspond to 1.5-fold change and adjusted p-value ≤0.05. **(F)** *Fx-mir* cluster schematic. MicroRNAs more highly expressed in BA2 are shaded orange and remaining microRNAs are shaded blue. **(G)** Z-score of overlapping or nearby protein-coding genes from BA RNA-seq (25). **(H)** Loci of microRNAs more highly expressed in the PBA/OFT overlapping Hox cluster gene members.

BA small-RNA-seq libraries were enriched for 18–25 nt reads (Supplementary Figure S1A), and 73%–86% of the 18–25 nt reads mapped to known microRNAs (Table 1). As shown by principal component analysis (PCA) (Figure 1A), replicate samples clustered closely to one another indicating reproducibility. The sample clustering across the first two principal components coincided with anterior-posterior location and developmental stage respectively. Using Spearman’s rank correlation to determine similarity between datasets, BA1 E11.5 and BA2 E11.5 showed the greatest correlation, followed by PBA/OFT E10.5 and PBA/OFT E11.5 (Supplementary Figure S1B). The PBA/OFT were most distinct in microRNA expression compared to the two anterior BA domains.

**TABLE 1:** Mapping of small-RNA-sequencing libraries.

Sample	Total reads	Filtered reads (rRNA/tRNA removed, 18–25 nt)	Filtered reads mapped to ≤ 5 genome locations	% Mapped reads assigned to microRNAs
BA1 E10.5 rep 1	34780431	24688368	22556923	82.30%
BA1 E10.5 rep 2	32326948	21955008	19878735	80.70%
BA1 E11.5 rep 1	6747799	5019435	4633059	84.30%
BA1 E11.5 rep 2	65348778	48884624	45278954	86.50%
BA2 E10.5 rep 1	40927309	30236945	26761729	78.10%
BA2 E10.5 rep 2	37812453	21752385	18847087	73.50%
BA2 E11.5 rep 1	29257674	20668574	18836846	82.40%
BA2 E11.5 rep 2	14788475	10,741,596	9668547	82.20%
PBA E10.5 rep 1	23692702	16826434	15047702	80.30%
PBA E10.5 rep 2	38,112,869	22309416	19913345	78.00%
PBA E11.5 rep 1	33688294	25154772	22616424	81.50%
PBA E11.5 rep 2	25473885	18359442	16464804	80.50%

Using miRDeep, and a set of *post hoc* filters for high confidence microRNA annotations, we identified 28 putative novel pre-microRNAs (Supplementary Table S2). The discovery of novel microRNAs is perhaps unexpected in such a well-studied model organism. However, it is well-known that many microRNAs show very specific special and temporal expression patterns (Xu et al., 2024), and therefore under-studied tissues and developmental time points continue to reveal novel microRNA loci. All these predicted loci pass accepted strict criteria, including for submission to the miRBase database (see methods), and as with all microRNAs, further datasets and studies will help to clarify these annotations. Two of the novel microRNAs share seed sequences with mmu-miR-702 and mmu-miR-1839.

After applying an expression cut-off (see methods), we found a total of 550 microRNAs were expressed across the BAs. We explored differential expression in both a spatial and temporal manner. To identify domain-specific microRNAs, we performed BA pairwise comparisons across E10.5 and E11.5 samples. We defined differentially expressed microRNAs as those with ≥1.5-fold change and adjusted p-value≤0.05. In at least one pairwise BA comparison for a given timepoint, 94 microRNAs were differentially expressed at E10.5, and 103 microRNAs were differentially expressed at E11.5. There was considerable overlap between the sets of differentially expressed microRNAs at E10.5 and at E11.5 (Figure 1B), showing that regional microRNA expression is largely maintained across these developmental timepoints. Additionally, at both E10.5 and E11.5, we saw increased microRNA expression progressively across the anterior-posterior axis, whereby most differentially expressed microRNAs demonstrated ≥1.5-fold expression in the PBA/OFT compared to BA1 and BA2 (Supplementary Figure S1C, D).

By considering the expression of microRNAs and their validated targets we can infer their regulatory outcome. For example, 11 mature microRNAs were significantly more highly expressed in BA2 compared to both BA1 and PBA/OFT at E10.5 (Figure 1C). These include miR-743b-3p, miR-741-3p, miR-878-5p, miR-881-3p, miR-871-3p, miR-470-5p, miR-465a/b/c-3p, and miR-465b/c-5p (Figures 1D, E). These microRNAs are transcribed from a large microRNA cluster, *Fx-mir*, spanning ∼62 kb on Chr X (Figure 1F). Members of this cluster have been described to regulate the neighbouring gene *Fmr1* (Ramaiah et al., 2019; Wang et al., 2020), however, each respective study draws contradictory conclusions with regards to whether these microRNAs repress or promote expression of *Fmr1*. Expression profiles from our BA RNA-seq show that *Fmr1* was most lowly expressed in BA2 (Figure 1G), suggesting *Fx-mir* microRNAs may have a repressive role on *Fmr1* in BA2.

The PBA/OFT demonstrated the most distinct microRNA expression across the BAs, at both E10.5 and E11.5 (Supplementary Figure S1C, D). Some of the greatest differentially expressed microRNAs (Figure 1E; Supplementary Figure S1E) are located within the Hox clusters; *miR-10b* overlaps with both *Hoxd3* and *Hoxd4*, *miR-10a* is located upstream of *Hoxb4*, and *miR-615* is found within *Hoxc5* (Figure 1H). These *Hox* genes are also more highly expressed in the PBA/OFT E10.5 (Figure 1G), coinciding with spatial collinear *Hox* gene expression in distinct streams of cranial NC cells that populate the BA’s (Frisdal and Trainor, 2014). Therefore, Hox cluster microRNAs in the PBA/OFT mirror expression of their overlapping or nearby protein-coding genes.

In summary, we have identified differentially expressed microRNAs across the BAs, and BA-specific expression patterns are largely maintained between E10.5 and E11.5. As E10.5 samples demonstrated more defined PCA clustering (Figure 1A) and had a greater number of differentially expressed microRNAs (Supplementary Figure S1C, D), we focused on this timepoint for the remainder of our study.

### 3.2 miR-92b-3p is a candidate regulator of cardiac developmental genes

**FIGURE 2:**
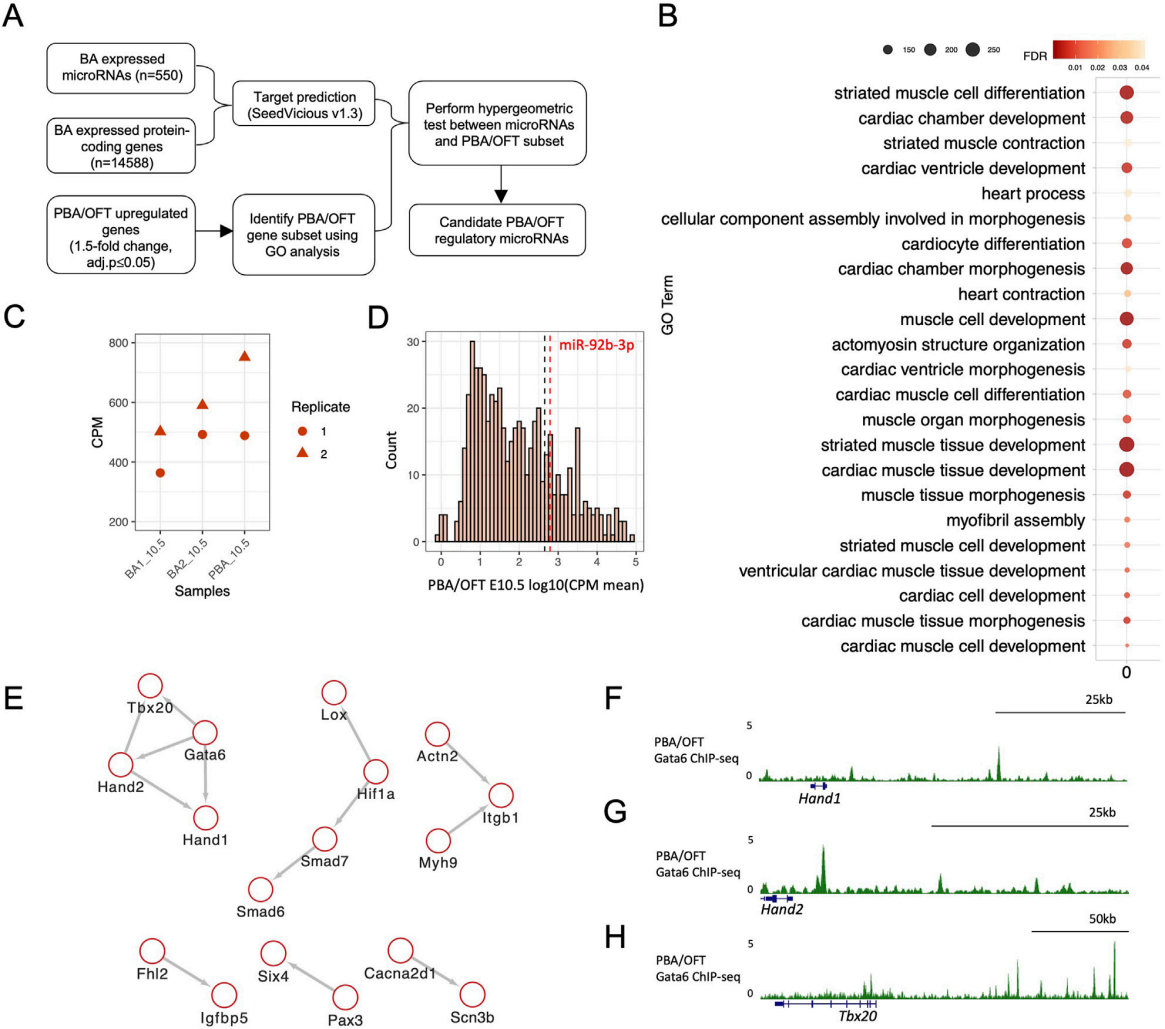
Identifying candidate microRNAs as regulators of PBA/OFT differentiation genes. **(A)** Pipeline for identifying candidate microRNA regulators of PBA/OFT differentiation. **(B)** GO analysis performed using PANTHER (Mi et al., 2018). Genes with ≥1.5-fold-change (adj-p ≤ 0.05) in the PBA/OFT, compared to both BA1 and BA2, from total RNA-seq were used as input, with all differentially expressed genes from BA pairwise comparisons used as background genes (fold-change ≥1.5, adj-p ≤ 0.05). Total RNA-seq was re-analysed from previous publication (25). **(C)** CPM of miR-92b-3p across BA1, BA2 and PBA/OFT at E10.5. **(D)** Log_10_(mean CPM) expression of all BA microRNAs in PBA/OFT E10.5. Black dashed line marks the 75^th^ percentile (2.66), red dashed line marks miR-92b-3p (log_10_ (meanCPM) = 2.79). **(E)** Protein-protein interaction network of predicted miR-92b-3p targets in the PBA/OFT gene subset, using the STRING database with a 0.6 confidence interaction score (Szklarczyk et al., 2021). **(F, G, H)** PBA/OFT E11.5 Gata6 ChIP-seq (mm 9) around predicted downstream targets of GATA6 (Losa et al., 2017).

The microRNA biogenesis pathway has previously been implicated in PBA artery remodelling (Nie et al., 2011) and OFT morphogenesis (Saxena and Tabin, 2010). We combined expression data from both microRNA-seq and RNA-seq datasets to identify individual microRNA mediated regulation in this domain. The approach is shown in Figure 2A. To begin, we generated a comprehensive list of predicted interactions between BA microRNAs and BA protein-coding genes (see methods). In parallel, we curated a subset of protein-coding genes specifically implicated in PBA/OFT development. We identified this subset by extracting genes that were more highly expressed in the PBA/OFT, relative to BA1 and BA2, performing GO analysis and selecting genes annotated under the top GO terms. Many of these terms were related to muscle and cardiac development, consistent with the contribution of this area to the anterior pole of the heart (Figure 2B). We obtained a list of 315 genes associated with developmental processes specific to the PBA/OFT. To identify microRNAs most significantly enriched for predicted interactions with these 315 genes, we performed a hypergeometric p-value test. For each microRNA, this test considers its predicted interactions with all BA genes and its predicted interactions with the 315 genes specific the PBA/OFT. In return, we determine the microRNAs that interact with this set of genes, compared to all BA genes as background, more than would be expected by chance.

The top ranked microRNA by hypergeometric p-value was miR-92b-3p (p-value = 0.0029). This microRNA was 1.7-fold enriched for predicted interactions with the PBA/OFT list of genes compared to interactions with all BA expressed mRNAs. On average, miR-92b-3p was most highly expressed in the PBA/OFT (Figure 2C) and expressed above the 75th percentile of all PBA/OFT microRNAs (Figure 2D). Looking at its predicted interactions, miR-92b-3p was predicted to bind to 30 of the 315 PBA/OFT genes (Supplementary Table S3).

MicroRNAs are understood to stabilise GRNs through broad regulation of multiple targets (Liufu et al., 2017). Therefore, we were interested in identifying interactions between miR-92b-3p predicted targets. To do so we used the STRING database, which returned evidence for multiple functional interaction networks (Figure 2E). One of these protein-protein interaction clusters contained four developmental cardiac TFs, GATA6, TBX20, HAND1 and HAND2, which have previously been shown to have correlated expression (Sharma et al., 2020). In human induced pluripotent stem cells (hiPSCs) undergoing cardiomyocyte differentiation, *HAND1, HAND2* and *TBX20* are downregulated following *GATA6* loss of function (Sharma et al., 2020). This may be a result of direct regulation, as GATA6 binds in the vicinity (100 kb upstream or downstream) of *Hand1*, *Hand2*, and *Tbx20* in the PBA/OFT (Losa et al., 2017) (Figures 2F–H).

Taken together, we generated *in silico* microRNA-target predictions using time-matched BA RNA-seq expression datasets. We identified miR-92b-3p as enriched for predicted binding sites for a network of cardiac transcription factors. As one of these transcription factors, *Hand2,* has previously been validated as a target of miR-92b-3p (Yu et al., 2019), we explored the possibility that miR-92-3p may regulate the other predicted targets *Gata6, Tbx20,* and *Hand1* to broadly modulate a cardiac GRN.

### 3.3 miR-92b-3p interacts with *Gata6* and *Tbx20* 3′UTRs

**FIGURE 3:**
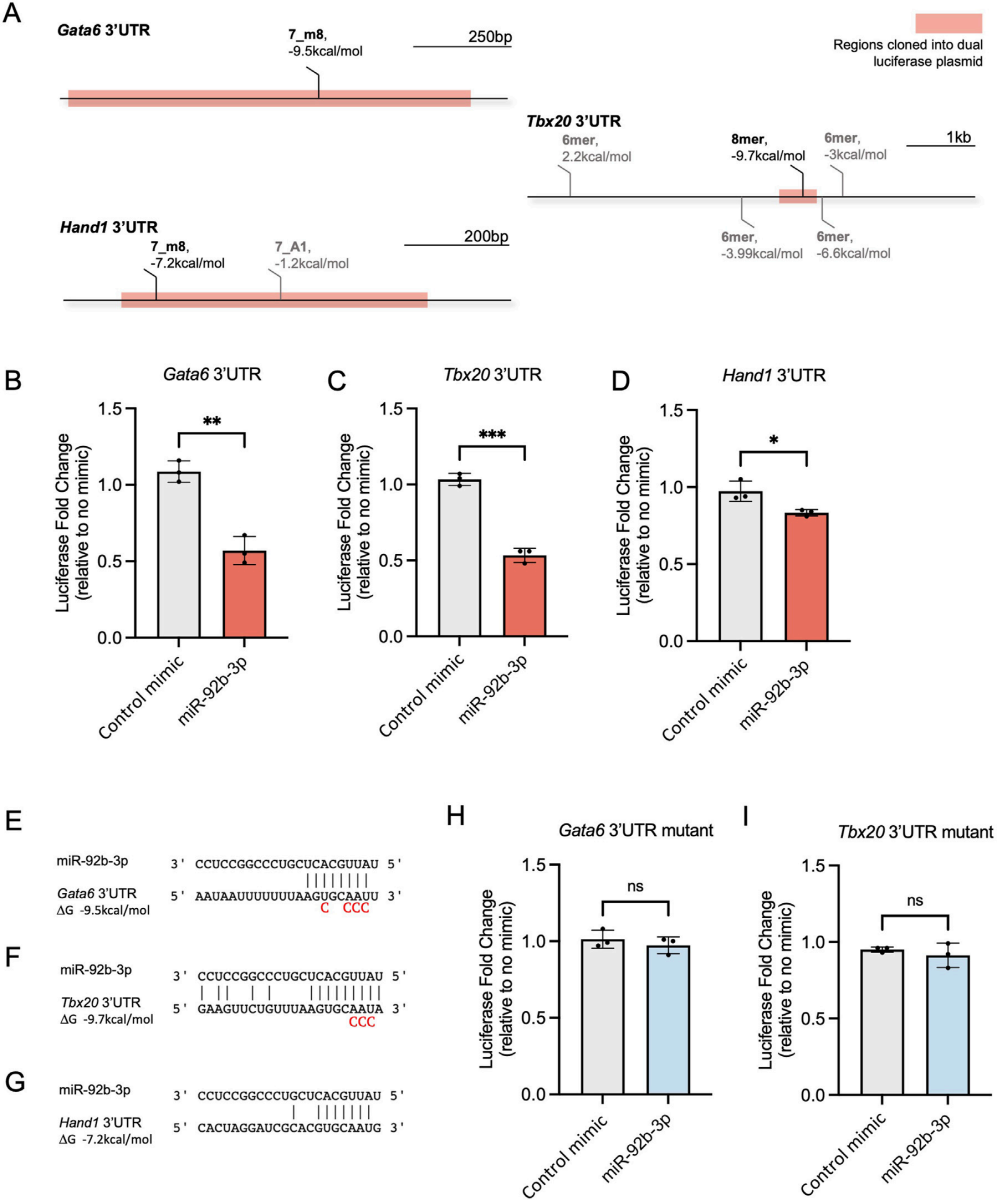
Testing miR-92b-3p binding sites in mouse Gata6, Tbx20, and Hand1 3′UTRs. **(A)** Seedvicious predicted binding sites between miR-92b-3p and Gata6, Tbx20, and Hand1 3’UTR’s. Highlighted regions were cloned into the pmirGLO dual-luciferase plasmid. Sites that did not meet our threshold of −7 kcal/mol are shown in grey. **(B–D)** Dual-luciferase reporter assays following co-transfection of microRNA mimics and dual luciferase plasmids containing wild type 3’UTR, in NIH/3T3 cells. Luciferase fold change is plotted relative to samples transfected with only plasmid and no mimic. Control mimic is mirVana negative control #1. Values are presented as the mean ± s.d, n = 3 biological replicates. Significance was calculated using an unpaired t-test: Gata6 p-value = 0.0015, Tbx20 p-value = 0.001, Hand1 p-value = 0.0254. **(E–G)** Predicted complementary binding and hybridisation energy between miR-92b-3p and Gata6, Tbx20, Hand1 3’UTRs. Mutated nucleotides included underneath wildtype sequences. **(H, I)** Dual-luciferase reporter assays following co-transfection of dual luciferase plasmids containing mutated 3’UTRs microRNA mimics. Values are presented as mean±s.d., n=3 biological replicates.

To assess whether miR-92b-3p can interact with its predicted target sites in *Gata6*, *Tbx20* and *Hand1* 3′UTRs, we used dual-luciferase reporter assays. Whilst we note these assays may not reflect physiological binding, our aim for this experiment was determine if binding can occur at the target sites and if this brings about reduced reporter expression. Portions of each 3′UTR containing the predicted binding sites were cloned into the Promega pmirGLO dual-luciferase plasmid (Figure 3A). Following co-transfection of miR-92b-3p mimic and *Gata6* and *Tbx20* 3′UTR reporters, we saw a significant reduction in luciferase signal, approximately 0.5-fold, compared to the control mimic (Figures 3B, C). In contrast, when miR-92b-3p was co-transfected with the *Hand1* 3′UTR reporter, a smaller reduction in reporter signal was seen compared to the control mimic (Figure 3D). These results are consistent with the predicted hybridisation energies between miR-92b-3p and the three putative binding sites (Figures 3E–G), whereby *Hand1* has the weakest interaction predicted at −7.2 kcal/mol, just meeting our −7 kcal/mol threshold cut-off.

Next, we determined whether luciferase reporter knockdown was specifically caused by the physical interaction of miR-92b-3p with the putative binding sites in *Gata6* and *Tbx20* 3′UTRs. Initial binding occurs between positions 2–4 of a microRNA seed site and the target RNA (Chandradoss et al., 2015), therefore, we mutated nucleotides complementary to the seed region of miR-92b-3p (Figures 3E, F). Following co-transfection of mutated 3′UTRs with miR-92b-3p, we did not observe reduced luciferase reporter expression, confirming luciferase reporter knockdown occurs due to interaction of miR-92b-3p with the identified binding sites located in *Gata6* and *Tbx20* 3′UTRs (Figures 3H, I).

In summary, miR-92b-3p interacts with the predicted target sites in *Gata6* and *Tbx20*, leading to significantly reduced reporter expression. Furthermore, the extent of repression was reflective of the predicted hybridisation energy.

### 3.4 miR-92b-3p binding to *Gata6* and *Tbx20* is conserved in human

#### 3.4.1 Conservation of miR-92b-3p binding sites across mouse and human

**FIGURE 4:**
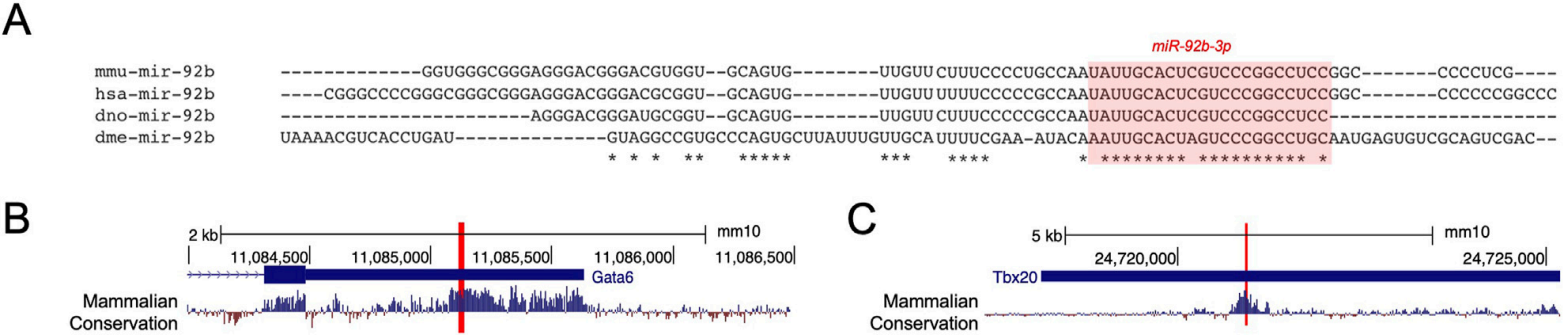
Conservation of miR-92b-3p targeting of *GATA6* and *TBX20*. **(A)** Alignment of miR-92b (3p highlighted) in *M. musculus*, *H. sapiens*, *D. rerio*, and *D. melanogaster*. **(B, C)** PhyloP placental mammalian basewise conservation in *Gata6* and *Tbx20* 3’UTRs. miR-92b-3p binding sites are highlighted in each 3’UTR.

Cardiac development is a highly conserved process (Jensen et al., 2013), and so we were interested in exploring whether there was conservation of miR-92b-3p binding sites within *Gata6* and *Tbx20* 3′UTRs. miR-92b-3p is conserved between species separated by more than 780 million years of evolution (Hedges et al., 2006). Alignment of pre-miR-92b across mouse, human, zebrafish, and *Drosophila* showed most conserved nucleotides are in the 3′ mature arm (Figure 4A). Additionally, the validated miR-92b-3p binding sites in mouse *Gata6* and *Tbx20* 3′UTRs are in regions of high sequence conservation (Kent et al., 2002) (Figures 4B, C, Supplementary Figure S2A, B). Consistent with these observations, *in silico* target prediction between human miR-92b-3p and *GATA6* and *TBX20* 3′UTRs identified homologous binding sites to those we validated by dual luciferase reporter assay, with identical seed complementarity and similar hybridisation energies (Supplementary Figure S2C, D). Therefore, we wanted to determine whether miR-92b-3p can regulate *GATA6* and *TBX20* in a similar repressive manner to that shown from our reporter assays, specifically in a human cardiac cell type.

#### 3.4.2 miR-92b-3p knocks down *GATA6* and *TBX20*

**FIGURE 5:**
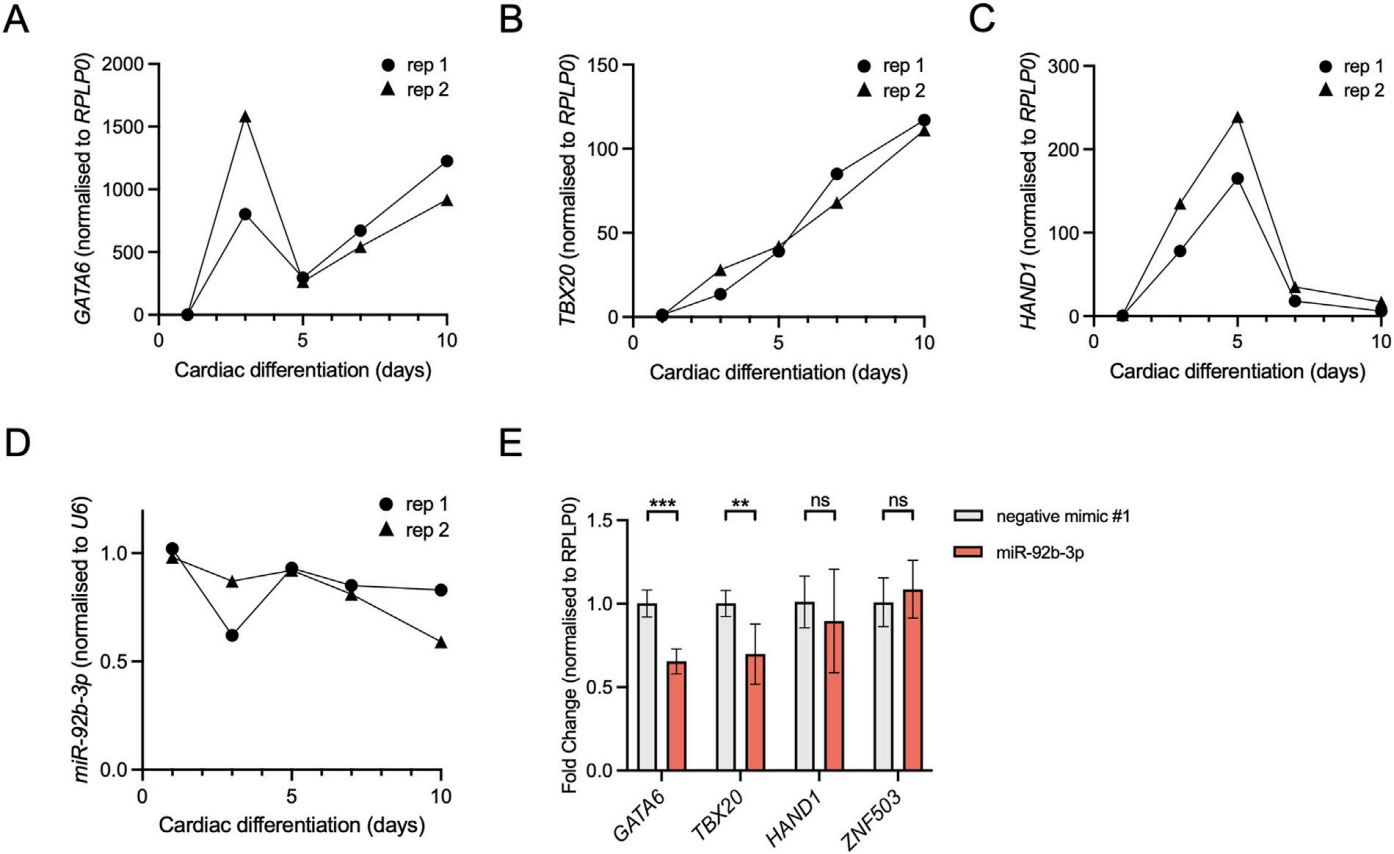
Knockdown of *Gata6* and *Tbx20* following miR-92b-3p transfection in a human cardiac cell type. **(A–C)** RT-qPCR of *GATA6*, *TBX20* and *HAND1* expression, normalised to *RPLP0*, during days 1–10 of hESC cardiomyocyte differentiation, n = 2 biological replicates. **(D)** miR-92b-3p expression, normalised to snRNA *U6*, during days 1–10 of a hESC cardiomyocyte differentiation, n = 2 biological replicates. **(E)** RT-qPCR of *GATA6*, *TBX20, HAND1,* and *ZNF503* (negative control), following 24 h transfection with miR-92b-3p mimic. hESCs were collected on day 7. Expression was normalised to *RPLP0* and then used to calculate fold change relative to the control mimic. Values are presented as the mean ± s.d., n = 6 biological replicates. Statistical significance was calculated performing multiple unpaired t-tests, *GATA6* adjusted p-value = 8.4 × 10^−5^, *TBX20* adjusted p-value = 4.6 × 10^−3^.

The PBA/OFT transcriptome is enriched for transcripts linked to cardiac muscle development and differentiation (Figure 2B). To test whether miR-92b-3p could regulate *GATA6* and *TBX20* in a cardiac cell type we employed a human cardiac differentiation system (Giacomelli et al., 2020). Firstly, we determined the expression dynamics of *GATA6, TBX20*, and miR-92b-3p within our model. We also included *HAND1,* to see how its expression pattern related to the other two transcription factors of interest. All 3 TFs were initially upregulated at the onset of differentiation, however, from day 3 onwards they displayed variable expression dynamics (Figures 5A–C). *GATA6* and *TBX20* expression increased between day 5 to day 10, whilst *HAND1* expression decreased. In contrast, we found miR-92b-3p remained generally stable throughout cardiac differentiation with a slight decrease in expression by day 10 (Figure 5D). We also measured miR-92b-3p in undifferentiated hESCs and found it to be expressed at similar levels as day 1 (data not shown), meaning its expression was not induced by differentiation. This is comparable to microarray data previously published (Wilson et al., 2010). Therefore, whilst not expressed in a cardiac-specific manner, miR-92b-3p is expressed at the same cardiac differentiation stages as *GATA6* and *TBX20* and could therefore function as a regulatory factor in a cardiac cell type.

To test whether miR-92b-3p can regulate endogenous *GATA6* and *TBX20,* we transfected differentiating cardiomyocyte cells with microRNA mimics and measured gene expression 24 h later. Following miR-92b-3p mimic transfection, *GATA6* and *TBX20* were both downregulated compared to the control mimic (Figure 5E), suggesting miR-92b-3p may downregulate their expression in a cardiac cell type. In contrast to *GATA6* and *TBX20*, we found no significant knockdown of *HAND1*, akin to results shown in Figure 3D, suggesting there is no functional effect of this miR-92b-3p predicted binding site. Additionally, a negative control gene *ZNF503* (with no predicted miR-92b-3p binding sites in its 3′UTR) did not display significant knockdown between conditions, demonstrating that miR-92b-3p transfection did not affect global transcription or translational machinery.

As *GATA6* loss of function has previously been shown to downregulate *TBX20* in a cardiac setting (Sharma et al., 2020)*,* the *TBX20* knockdown we observe could be an indirect effect, rather than a direct effect, of miR-92b-3p binding. To test whether miR-92b-3p reduces *TBX20* expression directly, rather than indirectly via GATA6 moderation, we performed microRNA transfection in HEK293 cells. This human cell line showed no evidence of GATA6-mediated *TBX20* regulation, as demonstrated by 1000-fold *GATA6* overexpression and no measurable changes to *TBX20* (Supplementary Figure S3A). We therefore transfected miR-92b-3p into HEK293 cells and measured endogenous *GATA6* and *TBX20* (Supplementary Figure S3B). Both *GATA6* and *TBX20* were significantly knocked down following miR-92b-3p transfection. Furthermore, we found reduced GATA6 expression, showing that miR-92b-3p repression extended to protein abundance (Supplementary Figure S3C). We did not see any significant difference in our negative control, ACTB, for both mRNA and protein, showing that overexpression of miR-92b-3p is unlikely to disrupt global transcription and translation.

To summarise, we have shown evidence of miR-92b-3p mediated repression of *GATA6* and *TBX20* in two human cell lines; one that provided a cardiac cell context, and one that provided a system for which *GATA6* and *TBX20* expression were independent of one another. We understand that there are limits to interpreting results from overexpression of microRNA mimics. Due to the excess in which microRNA mimics are applied to cells, they do not represent physiological microRNA expression levels. However, our work has concluded that miR-92b-3p can bind to both *Gata6* and *Tbx20* via the 3′UTR binding sites highlighted in this study. These binding sites are conserved between mouse and human, and downregulation of endogenous human *GATA6* and *TBX20* were akin to results shown by dual luciferase reporter assays. If miR-92b-3p-mediated regulation occurs at physiological levels, this would place miR-92b-3p within a cardiac GRN containing Gata6, Tbx20, and Hand2 (Yu et al., 2019).

## 4 Discussion

In this study we present novel small-RNA-seq datasets characterising microRNA expression across developing mammalian BAs as they undergo tissue specification and morphological changes. These libraries complement our previously published work on RNA-seq and ChIP-seq datasets (Amin et al., 2015; Donaldson et al., 2012; Losa et al., 2017), expanding our understanding of BA developmental biology into the microRNA field. As these small-RNA-seq datasets provide microRNA expression across all BA domains, these additionally build upon microRNA microarray data generated from isolated NC cells in mouse BA1 (Sheehy et al., 2010). Surprisingly, none of the NC-upregulated microRNAs reported by Sheehy et al. (2010) demonstrated BA1-specific expression in our datasets, suggesting alternative groups of microRNAs are important for BA identity compared to distinct cell populations within the BAs.

We have characterised expression of 550 mature microRNAs in the BAs, with the most distinct domain being the PBA/OFT with regards to microRNA upregulation. Additionally, we identified miR-92b-3p as a candidate regulator of cardiovascular development in the PBA/OFT. We validated its interaction with *Gata6* and *Tbx20,* two central cardiac TFs (Lepore et al., 2006; Losa et al., 2017; Sharma et al., 2020; Takeuchi et al., 2005). Previously, knockout of the microRNA biogenesis factor *Dicer* led to abnormal OFT development, with progenitor cells failing to differentiate into smooth muscle cells (SMC) (Sheehy et al., 2010). Incidentally, Gata6 is sufficient to promote SMC differentiation (Losa et al., 2017), highlighting one of the many microRNA-target interactions that may support normal OFT development. Cardiac development is also understood to be sensitive to gene or protein dosage, echoed by the incidence of human congenital cardiac malformations (Hoffman et al., 2004). Therefore, it is understandable that cardiovascular development is in part controlled by microRNA-directed regulation, and that microRNA dysregulation can therefore have an impact on disease (Goren et al., 2012; Hu et al., 2017; Ventura et al., 2008; Zhao et al., 2007).

TFs have been described as microRNA target hubs, with microRNAs essentially “regulating the regulators” (Martinez and Walhout, 2009). Previously the cardiac TF *Hand2* was identified as a target of miR-92b-3p (Yu et al., 2019), consistent with our *in silico* target predictions. Both Yu et al (2019) and Hu et al. (2017) showed that Ang-II induced cardiomyocyte hypertrophy caused an increase in miR-92b-3p expression in neonatal mouse ventricular cells. Furthermore, overexpression of miR-92b-3p prevented the hypertrophic phenotype developing following Ang-II treatment, through targeting *Hand2* (Yu et al., 2019). Another cardiac TF, *Mef2d*, is targeted by miR-92b-3p (Chen et al., 2012; Hu et al., 2017). While we identified *Mef2d* in our predicted targets of miR-92b-3p, *Mef2d* was not annotated under any of the GO terms used to identify our PBA/OFT subset genes and therefore not included in our network.

**FIGURE 6:**
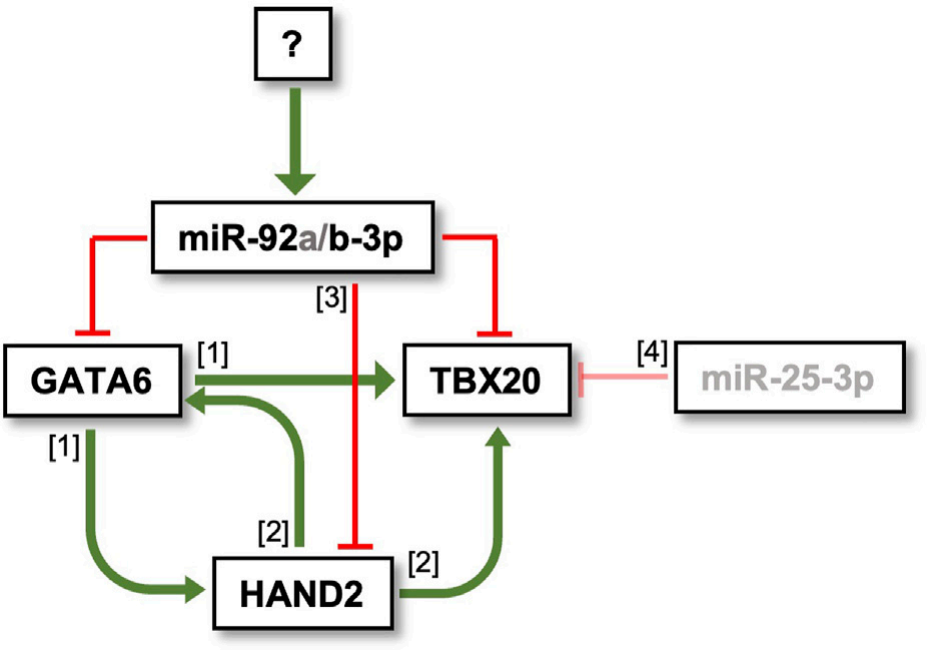
MicroRNA-mediated coherent feedforward loops which miR-92b-3p could regulate during PBA/OFT development. Regulatory interactions between TFs and microRNAs are supported by the following studies: [1] Sharma et al. (2020) [2] Laurent et al. (2017), [3] Yu et al. (2019), [4] Alzein et al. (2021).

The understanding of microRNA regulation within GRNs has advanced following the application of computational and mathematical modelling approaches (Cora’ et al., 2017; Lai et al., 2016). Different GRN motifs elicit different outputs (Lai et al., 2016), and it is therefore important to consider where a microRNA fits into a GRN to infer its functional role. GATA6, TBX20 and HAND2 are all cardiac progenitor markers and have important roles in activating cardiovascular cell fates (Lepore et al., 2006; Losa et al., 2017; Sharma et al., 2020; Takeuchi et al., 2005; Tsuchihashi et al., 2011). Furthermore, these 3 TFs demonstrate overlapping expression within the PBA/OFT, as shown by *in situ* hybridisation and fluorescence microscopy (Cai et al., 2011; Lepore et al., 2006; Losa et al., 2017; Vincentz et al., 2020). GATA6 indirectly promotes *HAND2* and directly promotes *TBX20* expression during hiPSC cardiomyocyte differentiation, through functioning as a pioneer cardiac factor (Sharma et al., 2020). Additionally, HAND2 binds to cis-regulatory modules associated with *Gata6* and *Tbx20* in embryonic hearts (Laurent et al., 2017). Taken together, this places miR-92b-3p in multiple microRNA-mediated coherent feedforward loops (Figure 6). Of note, the miR-92b-3p target sites in *Gata6*, *Tbx20* and *Hand2* 3′UTRs are all located in highly conserved regions, determined from the UCSC genome browser Multiz alignments of 60 vertebrates, suggesting a conserved regulatory network. MicroRNA-mediated coherent feedforward networks can function to minimise leaky transcripts or prevent spatial co-expression of the microRNA and its targets (Lai et al., 2016; Shalgi et al., 2009). From our microRNA-seq and RNA-seq datasets we know that miR-92b-3p, *Gata6* and *Tbx20* are all more highly expressed in the PBA/OFT domain. However, it would be interesting to determine at a greater resolution, for example, single-cell, whether miR-92b-3p is in fact inversely correlated with *Gata6, Tbx20* and *Hand2,* as may be expected if functioning to prevent spatial co-expression or minimise leaky transcripts (Lai et al., 2016).

Alternatively, microRNAs can also work as master regulators when embedded within coherent feedforward motifs (Cora’ et al., 2017). If the microRNA and target genes are expressed in the same cell, then the microRNA concentration is a controlling parameter, driven by competition for microRNA-target binding. The microRNA in turn can regulate and maintain the ratio of its targets relative to one another, ensuring stability in target concentration (Cora’ et al., 2017). This is particularly effective when one of the microRNA targets is a TF which regulates the other target (Riba et al., 2014), as we see in our proposed miR-92b-3p network, where GATA6 regulates *Tbx20.* We have evidence of *Gata6* and *Tbx20* co-expression in human embryonic and foetal OFT single-cell data (unpublished). Therefore, if miR-92b-3p were also co-expressed in these cells, microRNA-target competition could occur. As a result, miR-92b-3p could act to reinforce this GRN and facilitate PBA/OFT development in a “coordinate regulatory” manner (Liufu et al., 2017). To build on our understanding of miR-92b-3p within this GRN, it would be interesting to determine its upstream regulator.

MicroRNAs often act moderately to fine-tune their target gene expression, with the idea that “weak and broad” regulation is central to how microRNAs stabilise GRNs and contribute to developmental canalization (Alberti and Cochella, 2017; Liufu et al., 2017). However, this moderately repressive role often means there is no substantial phenotypic consequence when individual microRNAs are knocked out, as over 90% of microRNA activity is recognised as “weak” (Chen et al., 2019). There is also redundancy between microRNAs that share the same targets, therefore it is also important to consider how microRNAs may work collectively. miR-92b-3p belongs to a larger seed family of microRNAs containing miR-92a-3p, miR-25-3p, and miR-363-3p. Two of these, miR-92a-3p and miR-25-3p, were ranked in the top five candidates enriched for predicted binding sites in our PBA/OFT gene subset. As these microRNAs share identical seed sequences, there will likely be a high level of redundancy between their targets (Marco et al., 2012; Subasic et al., 2015). miR-92a-3p is located within the *miR-17-92* cluster, which has previously been linked to cardiomyocyte proliferation, hypertrophic cardiomyopathy, and aberrant cardiac ageing (Chen et al., 2013; Danielson et al., 2013; Zhang et al., 2012). Furthermore, deletion of this cluster caused ventricular septal defects in mouse models (Ventura et al., 2008). Another family member, miR-25, is expressed in the OFT and ventricular regions during embryonic chick development and is predicted to regulate *Tbx20* (Alzein et al., 2021). Evidence for miR-92b-3p regulated cardiac development extends to *Drosophila*, whereby miR-92b-3p exhibited muscle and cardiac specific expression (Chen et al., 2012). Taken together, we hypothesise that miR-92b-3p and members of its family, through cooperativity and redundancy, perform a central role in regulating the described cardiac GRN (Figure 6) during PBA/OFT development.

## Data Availability

The datasets presented in this study can be found in online repositories. The names of the repository/repositories and accession number(s) can be found in the article/Supplementary Material.
