# Supplementary material for: High-throughput microRNA sequencing in the developing branchial arches suggests miR-92b-3p regulation of a cardiovascular gene network
Source: Front Genet. 2025 Feb 20;16:1514925. doi: 10.3389/fgene.2025.1514925 (PMC11882518; doi:10.3389/fgene.2025.1514925)
Supplement: Supplementary file 2 [file DataSheet1.pdf]

Supplementary figure 1

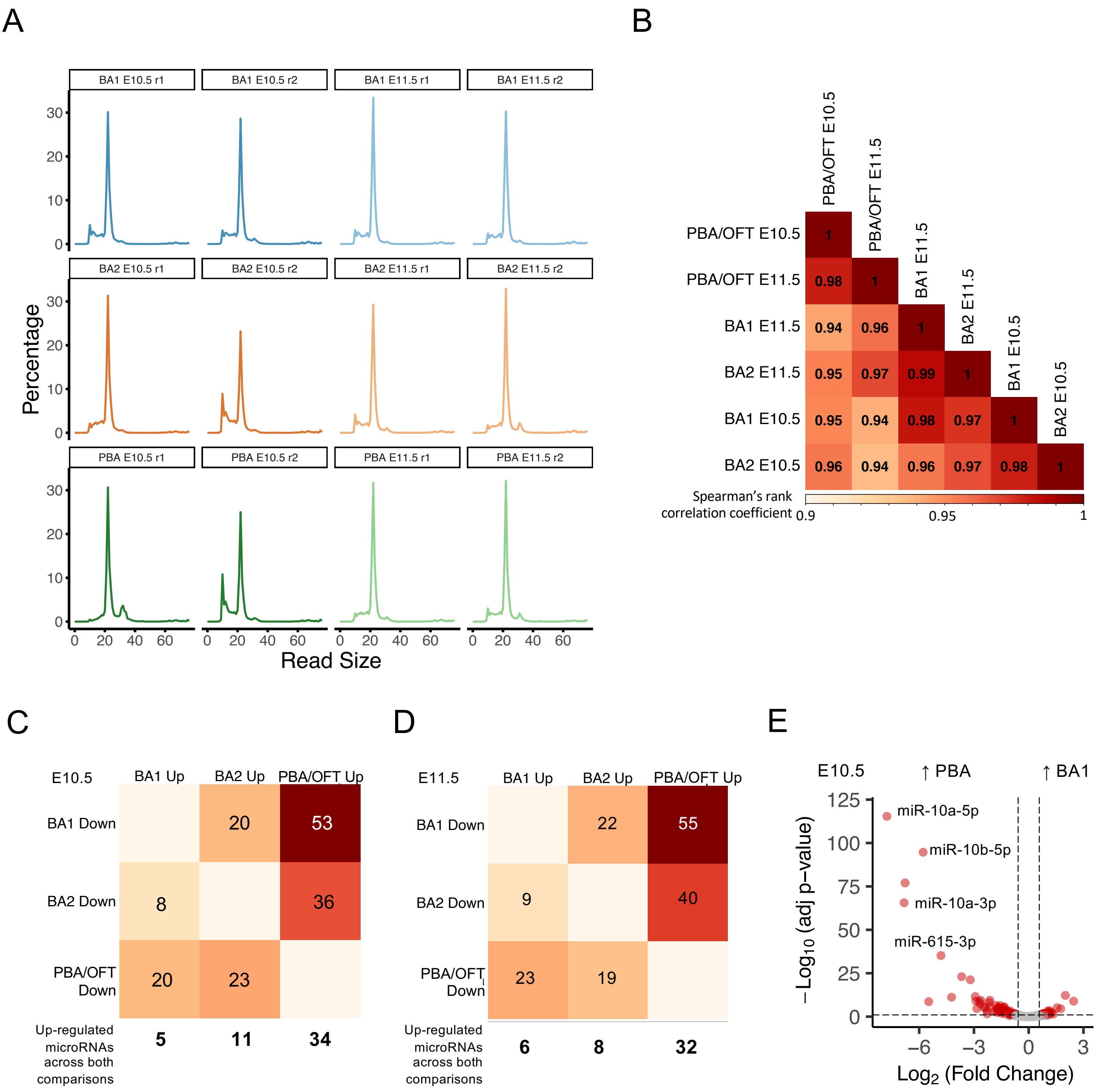

Supplementary Figure 1. BA small-RNA-seq library features.

(A) Read size across BA small-RNA seq libraries. (B) Spearman's rank correlation coefficient calculated using microRNA expression across BA samples. (C-D) Number of differentially expressed microRNAs across BA pairwise comparisons at E10.5 and E11.5. (E) Differentially expressed microRNAs at E10.5 in BA1 v PBA, with those located nearby PBA/OFT upregulated Hox genes labelled.

Supplementary figure 2

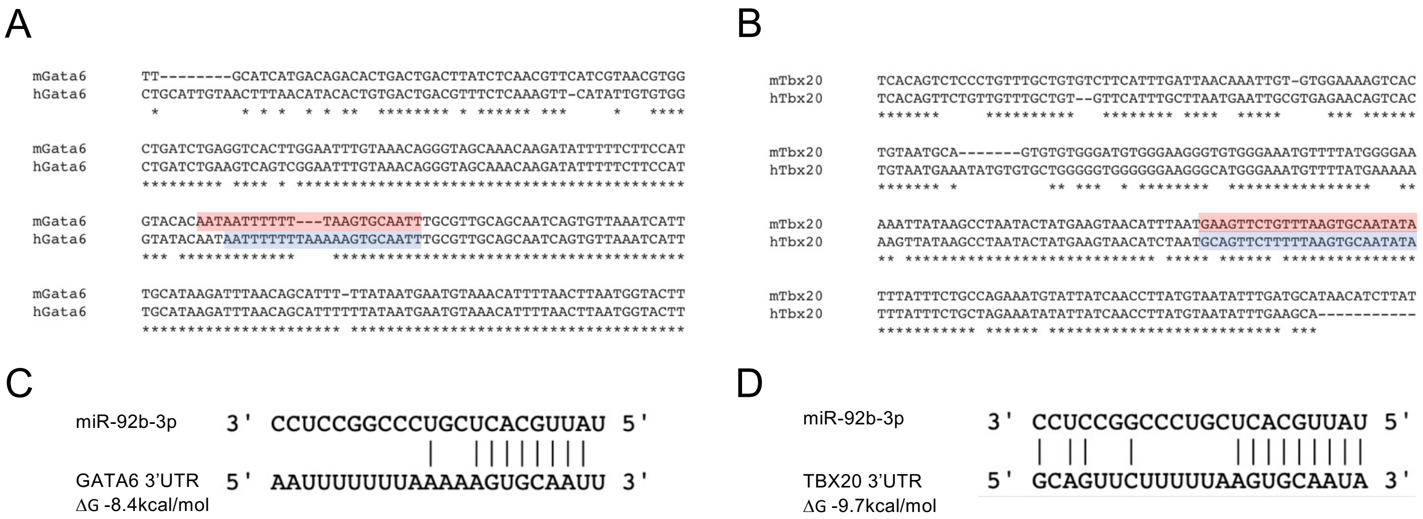

Supplementary Figure 2. Conservation of miR-92b-3p interaction with *GATA6* and *TBX20.*

(A, B) Alignment between mouse and human *Gata6/GATA6* and *Tbx20/TBX20* 3'UTRs surrounding the region with homologous miR-92b-3p binding sites. (C, D) Predicted interactions between miR-92b-3p and *GATA6* and *TBX20* 3'UTRs.

Supplementary figure 3

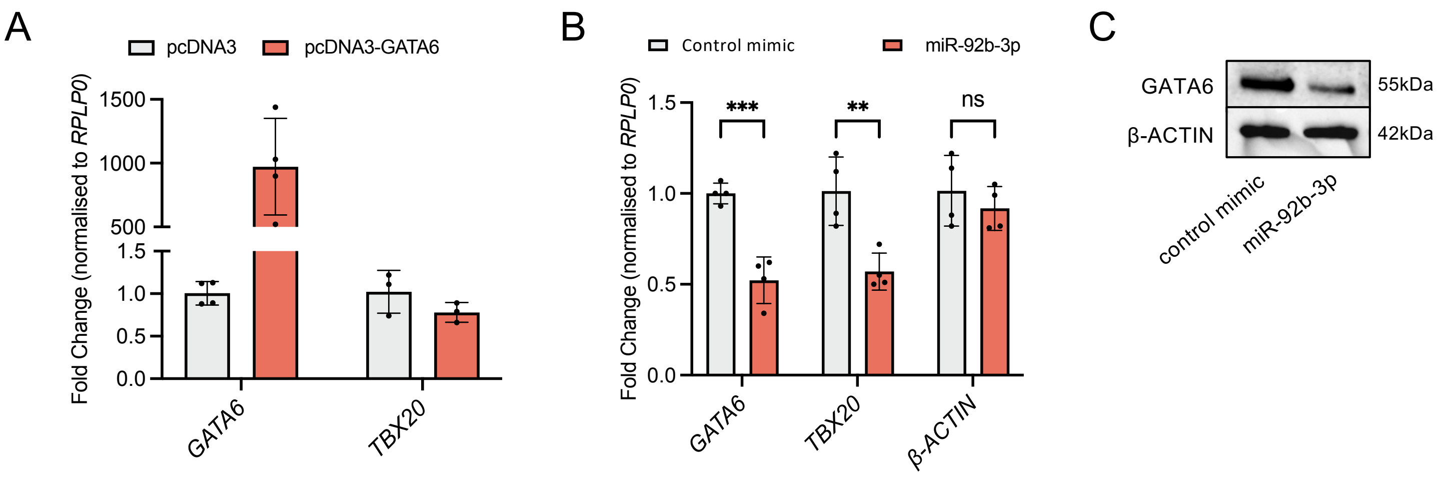

Supplementary Figure 3. miR-92b-3p transfection in HEK293 cells.

(A) *GATA6* and *TBX20* in HEK293 cells, 48h after transfection of pcDNA3-GATA6. Samples were first normalised to *RPLP0* and then used to calculate fold change. Values are presented as the mean±s.d., n=4 cultures. (B) *GATA6*, *TBX20,* and *ACTB* (negative control not predicted to be targeted by miR-92b-3p), in HEK293 cells 48h after transfection with 30nM microRNA mimics. Samples were normalised to *RPLP0* and then used to calculate fold change. Values are presented as the mean±s.d., n=4 cultures. Statistical significance was calculated performing multiple unpaired t-tests, *GATA6* p-value = 4.8×10^{-5}, *TBX20* p-value = 0.006. (C) Western blot of GATA6 and β-ACTIN, following 48h transfection with 30nM microRNA mimics. Replicate western blots showed similar levels of knockdown.

Supplementary File 1. List of primers

Primer	Sequence
NEBNext Index 1- BA1 E10.5 rep 1	CAAGCAGAAGACGGCATACGAGATCGTGATGTGACTGGAGTTCAGACGTGTGCTCTTCCGATCT
NEBNext Index 2- BA2 E10.5 rep 1	CAAGCAGAAGACGGCATACGAGATACATCGGTGACTGGAGTTCAGACGTGTGCTCTTCCGATCT
NEBNext Index 3- PBA E10.5 rep 1	CAAGCAGAAGACGGCATACGAGATGCCTAAGTGACTGGAGTTCAGACGTGTGCTCTTCCGATCT
NEBNext Index 4- BA1 E10.5 rep 2	CAAGCAGAAGACGGCATACGAGATTGGTCAGTGACTGGAGTTCAGACGTGTGCTCTTCCGATCT
NEBNext Index 5- BA2 E10.5 rep 2	CAAGCAGAAGACGGCATACGAGATCACTGTGTGACTGGAGTTCAGACGTGTGCTCTTCCGATCT
NEBNext Index 6- PBA E10.5 rep 2	CAAGCAGAAGACGGCATACGAGATATTGGCGTGACTGGAGTTCAGACGTGTGCTCTTCCGATCT
NEBNext Index 7- BA1 E11.5 rep 1	CAAGCAGAAGACGGCATACGAGATGATCTGGTGACTGGAGTTCAGACGTGTGCTCTTCCGATCT
NEBNext Index 8- BA2 E11.5 rep 1	CAAGCAGAAGACGGCATACGAGATTCAAGTGTGACTGGAGTTCAGACGTGTGCTCTTCCGATCT
NEBNext Index 9- PBA E11.5 rep 1	CAAGCAGAAGACGGCATACGAGATCTGATCGTGACTGGAGTTCAGACGTGTGCTCTTCCGATCT
NEBNext Index 10- BA1 E11.5 rep 2	CAAGCAGAAGACGGCATACGAGATAAGCTAGTGACTGGAGTTCAGACGTGTGCTCTTCCGATCT
NEBNext Index 11- BA2 E11.5 rep 2	CAAGCAGAAGACGGCATACGAGATGTAGCCGTGACTGGAGTTCAGACGTGTGCTCTTCCGATCT
NEBNext Index 12- PBA E11.5 rep 2	CAAGCAGAAGACGGCATACGAGATTACAAGGTGACTGGAGTTCAGACGTGTGCTCTTCCGATCT
Gata6-3'UTR forward	TAAGCAGAGCTCGAGCTGGTGCTACCAAGAGG
Gata6-3'UTR reverse	TAAGCAGTCGACGGTACAGGCGTCAAGAGTGT
Gata6-3'UTR mutagenesis forward	GTACACAATAATTTTTTTAAGCGCCCCTTGCGTTGCAGCAATCAGTG
Gata6-3'UTR mutagenesis reverse	CACTGATTGCTGCAACGCAAGGGGCGCTTAAAAAAATTATTGTGTAC
Tbx20 3'UTR forward	TAAGCAGAGCTCTCACAGTCTCCCTGTTTGCTG
Tbx20 3'UTR reverse	TAAGCAGTCGACCAGGTGAGAATATACAGACACCAGA
Tbx20 3'UTR mutagenesis forward	GTTTAAGTGCCCCATATTTATTTCTGCCAGAAATGTATTATCAAC
Tbx20 3'UTR mutagenesis reverse	GTTGATAATACATTTCTGGCAGAAATAAATATGGGGCACTTAAAC
Hand1-3'UTR forward	TAAGCAGAGCTCGCGGTTGCAACTACCCACTA
Hand1-3'UTR reverse	TAAGCAGTCGACCAGCAACGAATGGGAACGC
GATA6 RT-qPCR forward	CCACAACACAACCTACAGCC
GATA6 RT-qPCR reverse	ACGCCTATGTAGAGCCCATC
TBX20 RT-qPCR forward	GAGGGAAAGTGTGGAGAGCC
TBX20 RT-qPCR reverse	AAGGCTGACCCTCGATTTGG
HAND1 RT-qPCR forward	TCTTCCACCCTTTTGGAGCG
HAND1 RT-qPCR reverse	GCCTTTCATCTTCCTGCGTC
RPLP0 RT-qPCR forward	CACCATTGAAATCCTGAGTGATGT
RPLP0 RT-qPCR reverse	TGACCAGCCCAAAGGAGAAG
ZNF503 RT-qPCR forward	GCACGAACTGGCCACATTTT
ZNF503 RT-qPCR reverse	GGGCTCTTCTTGGCATCGAG
ACTB RT-qPCR forward	GGCTGTATTCCCCTCCATCG
ACTB RT-qPCR reverse	CCAGTTGGTAACAATGCCATG
pmirGLO sequencing primer forward	GGCAAGATCGCCGTGTAATTC
pmirGLO sequencing primer reverse	TATCATGTCTGCTCGAAGCG

Supplementary File 2. High-confidence novel mouse microRNAs predicted using miRDeep2 (Friedländer et al., 2011)

**miRDeep2 microRNA ID**	**Shared seed with mouse**	**Mature sequence [1]**	**Star sequence [2]**	**Genomic location**	**Relative location**
chr1_2334	-	uguguguaaggguccugucaau	ugugcagguccuguacauucagug	chr1:106669442..106669504:-	*Bcl20*, intron
chr2_6424	-	agcagcagcuggagcagugggggc	ccucuccuccggcggccgcggc	chr2:153444424..153444481:-	*Nol4l*, intron
chr2_5073	-	ucaaggucuagcagguagcacagc	gugagugccugcugcccugaguu	chr2:181379689..181379758:+	*Zgpat*, exon
chr2_3301	-	uauucugacucuaccuaacugga	ucugcuagguagugguggaacugc	chr2:18177241..18177305:+	*Mllt10*, intron
chr3_9116	-	cugagccccgagacuguauuac	uuacaguccgagcccugagacu	chr3:121770700..121770760:-	*Abcd3*, intron
chr5_14235	-	uaugaguucuaggcauugaaucu	gucaaugcucugaacuccuaag	chr5:33618090..33618153:-	*Fam53a*, intron
chr7_20796	-	ugacucucugcuuccccccagu	uuaggggguugcugagggaccu	chr7:126375202..126375257:-	*Spns1*, intron, <= 10kb miR-7058
chr8_23232	-	uguccgggggaccgacuuugcc	auggagccgcgcuccgggacgcgu	chr8:105496918..105496972:-	intergenic
chr8_22037	-	ucagccguucagcaccguccagaca	acugggcaaggaacaggcucggg	chr8:105660297..105660358:+	*Ctc*f, intron
chr8_22488	-	aaacugucugucuguucauagc	cauaugagcagaccuccaguuu	chr8:13885880..13885935:-	*Coprs,* intron
chr9_24954	*mmu-miR-1839-5p*	uagguagaccaggcugaucu	auccuccugucucugccuucu	chr9:122981876..122981935:+	*Kif15*, intron
chr9_23920	-	ccaggcugcuggagucugggu	ccagucuccaucugccuuccu	chr9:40696225..40696280:+	*Clmp*, intron
chr9_25372	-	ucagacugcugagucacauugc	aaugugacucagcuaccugaac	chr9:52103707..52103764:-	*Gm27686*, exon
chr9_24142	*mmu-miR-702-5p*	gugaguggagacucgguagaggu	ccucuccuguucuccaucuagu	chr9:57141915..57141995:+	*Man2c1*, intron
chr9_25805	-	aaaagaacugcguggcuaucuggc	ugagguagucagcagugaucuuu	chr9:94520299..94520363:-	*Dip2ka*, 3'UTR
chr10_26210	-	gagggacauacucaaugagaac	ucuuauuguccaugucccugcc	chr10:4092813..4092871:+	*Mthfd1l*, intron
chr11_30225	-	uccugccccuuucccuguaga	uaaggguggaugggcagagcu	chr11:105347182..105347235:+	*Mrc2*, intron
chr11_28554	-	uggcugccagcagagccuggau	ccacucugcugggcagcccag	chr11:4675148..4675209:+	*Ascc2*, intron
chr11_30626	-	ucuguuggauccugugaggaca	cccauaggauuuuaacagaacu	chr11:5080868..5080921:-	*Ewsr1*, intron
chr11_31180	-	uagcguucucggagaucauga	ucugaugacugagauugcugacc	chr11:61546572..61546631:-	*Epn2*, exon
chr11_30658	-	cuugaucuucucccucugcagg	ugcggaaggacagauccugugg	chr11:6264032..6264090:-	*Ddx56*, intron
chr12_33597	-	ucaagugugacaagaucucuac	ugaguuaucuugaggcacuugac	chr12:13245067..13245126:-	*Ddx1*, intron
chr12_32939	-	ugcugaauccagagguuacaucu	uggugaucucggagauucagg	chr12:73284657..73284720:+	*Trmt5*, antisense exon
chr15_39753	-	aauagcuugggacaucagcucugu	agaacugaugacugagcaagg	chr15:36595713..36595768:-	*Pabpc1*, 3'UTR
chr16_41858	-	ucaugugucucuuguguugauc	ccggcacaacagaaacaugagu	chr16:44490355..44490412:-	*Boc*, intron
chr16_41185	-	aacauguuggacggugcacu	augcgcugucagucaacauggc	chr16:64858038..64858103:+	*Cggbp1*, 3'UTR
chr19_47164	-	ugucuugggcucuggaguugaguu	aucgcugcugagccuagacugg	chr19:45773947..45774009:-	*Oga*, intron
chr19_46558	-	ugggcuccgccugugguccgc	cugacuuccaggcccagcccugca	chr19:57467284..57467344:+	*Trub1*, intron

Supplementary File 3. miR-92b-3p PBA/OFT subset target predictions.

miRNA	gene_name	position	type	value	3'UTRlength	seed
MIMAT0004899	ENSMUSG00000000631	711	7_m8	-7.4	1148	AUUGCA
MIMAT0004899	ENSMUSG00000004872	1365	8mer	-11.1	2041	AUUGCA
MIMAT0004899	ENSMUSG00000005836	665	7_m8	-9.5	1156	AUUGCA
MIMAT0004899	ENSMUSG00000008136	304	7_m8	-9.5	443	AUUGCA
MIMAT0004899	ENSMUSG00000021109	546	7_A1	-9.2	1830	AUUGCA
MIMAT0004899	ENSMUSG00000022443	579	7_m8	-10.7	1315	AUUGCA
MIMAT0004899	ENSMUSG00000022803	483	7_A1	-8.2	608	AUUGCA
MIMAT0004899	ENSMUSG00000024529	1074	7_m8	-7.8	3163	AUUGCA
MIMAT0004899	ENSMUSG00000024593	3397	7_A1	-9.3	3415	AUUGCA
MIMAT0004899	ENSMUSG00000025809	999	7_m8	-7.64	2529	AUUGCA
MIMAT0004899	ENSMUSG00000025809	1080	7_m8	-12	2529	AUUGCA
MIMAT0004899	ENSMUSG00000025880	1387	7_m8	-8	1573	AUUGCA
MIMAT0004899	ENSMUSG00000026185	3674	7_m8	-8.6	4448	AUUGCA
MIMAT0004899	ENSMUSG00000027474	1267	7_A1	-7.2	1643	AUUGCA
MIMAT0004899	ENSMUSG00000027887	2905	7_A1	-8.1	3067	AUUGCA
MIMAT0004899	ENSMUSG00000030790	299	7_m8	-9.4	655	AUUGCA
MIMAT0004899	ENSMUSG00000031965	4538	8mer	-9.7	7317	AUUGCA
MIMAT0004899	ENSMUSG00000034460	784	7_A1	-8.1	5173	AUUGCA
MIMAT0004899	ENSMUSG00000036867	209	7_m8	-9.3	451	AUUGCA
MIMAT0004899	ENSMUSG00000037335	184	7_m8	-7.2	897	AUUGCA
MIMAT0004899	ENSMUSG00000038193	128	7_m8	-9.87	776	AUUGCA
MIMAT0004899	ENSMUSG00000040118	1078	7_m8	-9.3	3840	AUUGCA
MIMAT0004899	ENSMUSG00000041842	899	7_m8	-7.59	2272	AUUGCA
MIMAT0004899	ENSMUSG00000042942	1650	8mer	-10.84	2460	AUUGCA
MIMAT0004899	ENSMUSG00000044447	782	8mer	-9.1	4286	AUUGCA
MIMAT0004899	ENSMUSG00000045092	1248	7_m8	-10.19	1365	AUUGCA
MIMAT0004899	ENSMUSG00000049281	1555	8mer	-7.8	3184	AUUGCA
MIMAT0004899	ENSMUSG00000052374	41	7_A1	-9.6	71	AUUGCA
MIMAT0004899	ENSMUSG00000055022	1375	7_m8	-14.9	2381	AUUGCA
MIMAT0004899	ENSMUSG00000062991	901	7_m8	-8.3	3480	AUUGCA
MIMAT0004899	ENSMUSG00000063632	1386	8mer	-7.74	6960	AUUGCA
